# Case report: Usefulness of angioscopy in determining antiplatelet drug reduction after carotid artery stenting

**DOI:** 10.3389/fneur.2023.1152173

**Published:** 2023-09-04

**Authors:** Kenji Fukutome, Mikio Shiba, Shuta Aketa, Takaaki Mitsui, Yuki Shiraishi, Hiromichi Hayami, Yasutaka Murakami, Ryuta Matsuoka, Rinsei Tei, Yasushi Shin, Yasushi Motoyama

**Affiliations:** ^1^Department of Neurosurgery, Osaka Police Hospital, Osaka, Japan; ^2^Cardiovascular Division, Osaka Police Hospital, Osaka, Japan; ^3^Department of Neurology, Osaka Police Hospital, Osaka, Japan

**Keywords:** angioscopy, angiography, carotid artery stenting, dual antiplatelet therapy, neointima

## Abstract

We report a case in which neointima was confirmed by angioscopy and antiplatelet drug administration was reduced 2 months after carotid artery stenting (CAS). A patient in their 80s was scheduled to undergo resection for renal cancer; however, he also had right cervical internal carotid artery stenosis. Because this was a risk for general anesthesia, CAS was performed after first starting dual antiplatelet therapy. Urologically, early reduction of antiplatelet drugs was necessary for a nephrectomy. Although no obvious neointima could be identified on ultrasound 2 months after CAS, thin neointima was observed using angioscopy. Based on the above results, we reduced the antiplatelet drug administration, and then the nephrectomy was performed. Ultimately, no cerebral infarction occurred in the perioperative or postoperative periods. Angioscopy allows for visual confirmation of thin neointima. If sufficient neointima can be confirmed, antiplatelet drug reduction can be performed more safely and reliably.

## Introduction

Before carotid artery stenting (CAS), dual antiplatelet treatment (DAPT) is necessary to prevent thrombosis (1–4). Long-term DAPT carries the risk of bleeding problems; thus, it is preferable to reduce the dose as soon as possible (3, 4). However, it is unclear how long it should be continued following CAS. If the struts of the stent are suitably coated with neointima, the risk of thrombosis is decreased, and antiplatelet medications may be stopped early. However, because early neointima is thin, it can be challenging to detect using carotid ultrasonography (CUS) or angiography. In this work, we describe a case in which CUS was unable to identify the neointima, but macroscopic inspection using angioscopy could detect the presence of a thin neointima, thereby allowing for a reduction in antiplatelet medication 2 months after CAS.

## Case report

**Figure 1:**
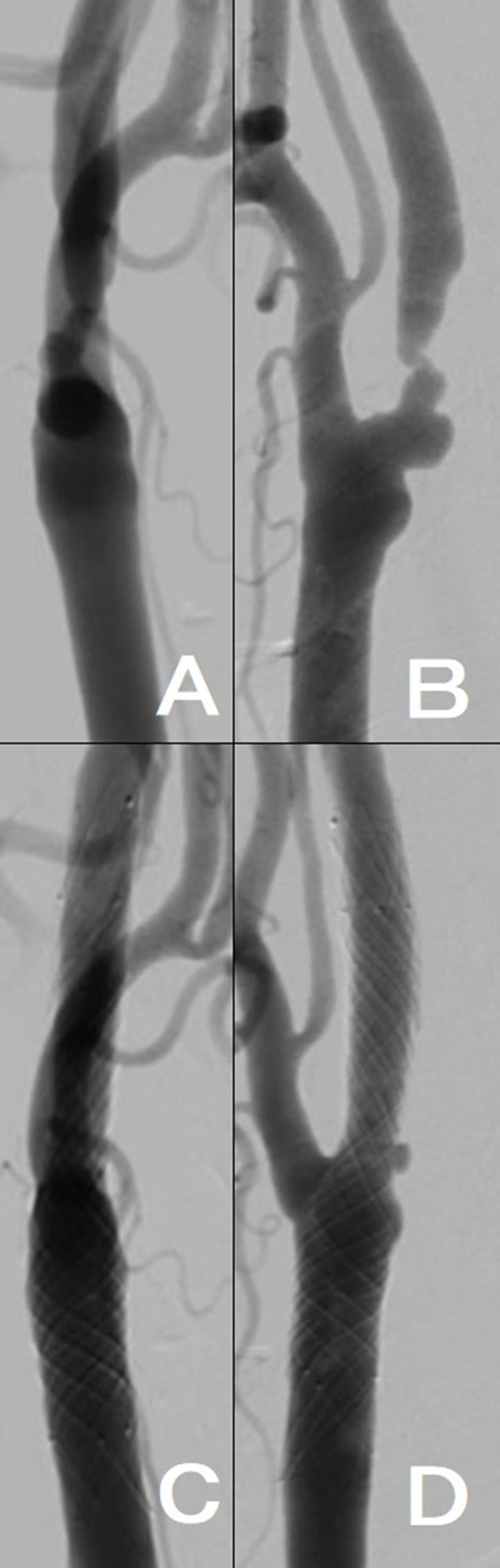
**(A,B)** Right internal carotid artery origin was 82% stenosed (North American Symptomatic Carotid Endarterectomy Trial method) according to the results of right common carotid artery angiography (ICA). **(C,D)** CASPER Rx was placed at the right ICA; the blood flow improved. [**(A,C)**, anteroposterior view; **(B,D)**, lateral view].

A patient in their 80s was scheduled to undergo resection for renal cancer, but he was referred to our department because right cervical internal carotid artery stenosis was suddenly discovered when using CUS for screening before general anesthesia. Right common carotid artery angiography revealed 82% stenosis (North American Symptomatic Carotid Endarterectomy Trial method), even though it was asymptomatic (Figures 1A,B); therefore, after beginning DAPT (aspirin and clopidogrel), CAS utilizing CASPER Rx (Terumo, Tokyo, Japan) was conducted (Figures 1C,D). In terms of urology, an early nephrectomy was required along with a reduction in antiplatelet medication.

**Figure 2:**
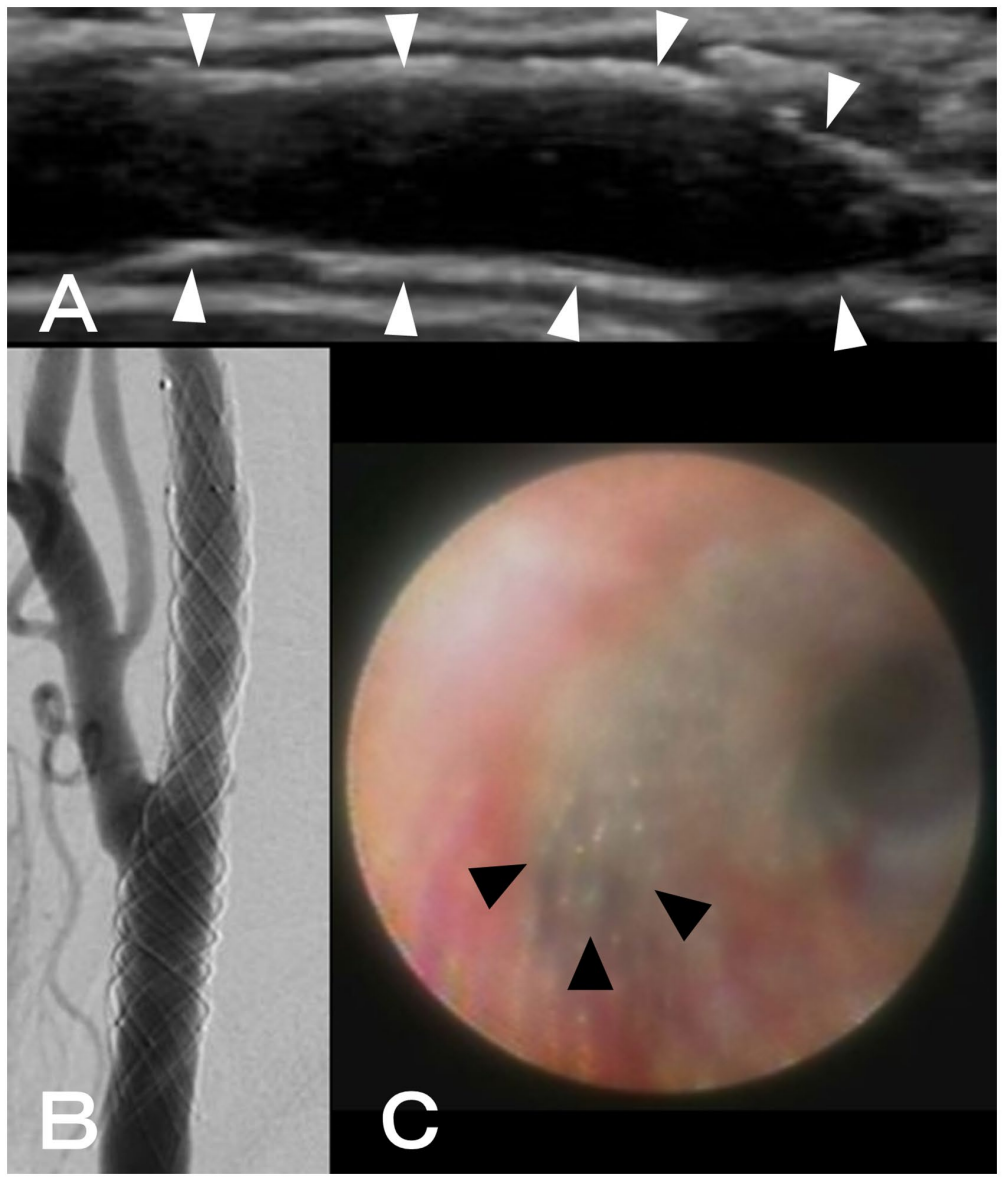
**(A)** Using ultrasonography, neointimal formation in CASPER Rx could not be detected (white arrowheads: CASPER Rx). **(B)** A radiolucent gap between the stent and artery lumen was discovered during right common carotid angiography. **(C)** Using angioscopy, thin neointimal development was seen throughout, except for the external carotid artery orifice (black arrowheads: the orifice of the external carotid artery).

Two months after CAS, another workup with angiography and angioscopy was conducted because it was challenging to detect the neointima within the stent using CUS (Figure 2A). After systemic heparinization, right common carotid angiography showed a radiolucent gap between the stent and lumen of the artery, suggesting neointimal formation (Figure 2B). After that, thin neointimal development was observed throughout the stent, except for the external carotid artery orifice, where the lumen was visually inspected with an angioscope VISIBLE (Intertec Medicals, Osaka, Japan) under proximal blood flow obstruction with a balloon catheter (Figure 2C). The obstruction time was less than 1 min and there were no ischemic symptoms.

Based on the above findings, we determined that the risk of thrombosis was low, decreased the antiplatelet drug administration to one (only aspirin), and then conducted the nephrectomy at the department of urology. In the end, there was no perioperative or postoperative cerebral infarction.

## Discussion

Although there are accounts of angioscopy evaluations of plaque before and after (5–7), there are no reports of angiography evaluations of neointima sometime after CAS. In this report, CUS was conducted 2 months after CAS, but no neointima could be identified. However, investigations on coronary arteries have indicated that neointima are typically seen 1–1.5 months following the implantation of a bare-metal stent (8). Although it is challenging to decide whether or not to reduce the antiplatelet drug administration based on the findings of CUS alone, direct confirmation of the neointima by angioscopy may allow a safer and more reliable decision to reduce the dose. Other intravascular ultrasound devices exist, such as intravascular ultrasound and optical coherence tomography; however, similar to CUS, identification is problematic if the neointima is thin. In general, neointima may be formed more or less 2 months after CAS, and it is possible that antiplatelet medication can be lowered or stopped at this point. The timing of neointima development may vary if a stent with a different shape, such as a single layer, is employed over a dual layer stent, such as the one used in this study. On the one hand, a dual layer is less likely to form neointima because of the increased amount of metal; on the other hand, it is more likely to form neointima because of the increased scaffolding.

A drawback of an angioscope is that, depending on the blood vessel’s diameter, it is rigid and has a narrow field of vision, making it challenging to thoroughly inspect the interior of a stent. It is a somewhat more intrusive test than conventional angiography because proximal blood flow blockage is required to provide a decent viewing field.

## Conclusion

Thin neointima that is not visible under CUS can be visually confirmed using angioscopy. Antiplatelet drug lowering can be conducted more securely and consistently if enough neointima can be verified. Antiplatelet medication may typically be lowered or stopped at that point because in-stent neointima formation is complete 2 months after CAS.

## Data availability statement

The original contributions presented in the study are included in the article/Supplementary material, further inquiries can be directed to the corresponding author.

## Ethics statement

The studies involving human participants were reviewed and approved by Osaka Police Hospital. The patients/participants provided their written informed consent to participate in this study. Written informed consent was obtained from the participant/patient(s) for the publication of this case report.

## Author contributions

KF, MS, SA, TM, YuS, HH, YMu, RM, RT, YaS, and YMo contributed to the work described in this paper, involved in the clinical management of the patient, and revised the manuscript. KF and MS conceived and designed the experiment, and drafted the manuscript. SA and YM supervised and coordinated the study and the manuscript. All authors contributed to the article and approved the submitted version.

## Conflict of interest

The authors declare that the research was conducted in the absence of any commercial or financial relationships that could be construed as a potential conflict of interest.

## Publisher’s note

All claims expressed in this article are solely those of the authors and do not necessarily represent those of their affiliated organizations, or those of the publisher, the editors and the reviewers. Any product that may be evaluated in this article, or claim that may be made by its manufacturer, is not guaranteed or endorsed by the publisher.
